# Career and education outcomes among people who acquired HIV perinatally and received care at two tertiary care hospitals in Bangkok, 2014–2020

**DOI:** 10.1371/journal.pone.0348339

**Published:** 2026-08-25

**Authors:** Rangsima Lolekha, Piyarat Suntarattiwong, Vitharon Boon-yasidhi, Yossawadee Na-Nakorn, Yuitiang Durier, Warunee Punpanich Vandepitte, Sanny Chen Northbrook, Chuenkamol Sethaputra, Wanatpreeya Phongsamart, Vorapathu Thaineua, Kulkanya Chokephaibulkit, on behalf of the Happy Teen Working Group

**Affiliations:** 1 Division of Global HIV and Tuberculosis, U.S. Centers for Disease Control and Prevention Thailand Office, Nonthaburi, Thailand; 2 Divisions of Infectious Disease, Department of Pediatrics, Queen Sirikit National Institute of Child Health, Ministry of Public Health, College of Medicine, Rangsit University, Bangkok, Thailand; 3 Department of Pediatrics, Faculty of Medicine Siriraj Hospital, Mahidol University, Bangkok, Thailand; 4 Siriraj Institute of Clinical Research, Faculty of Medicine Siriraj Hospital, Mahidol University, Bangkok, Thailand; Dr D Y Patil Vidyapeeth University Dr D Y Patil Medical College Hospital and Research Centre: Dr D Y Patil Medical College Hospital and Research Centre, INDIA

## Abstract

**Background:**

People born with HIV who receive antiretroviral treatment (ART) are living longer. Data on long-term education and employment among people who acquired HIV perinatally (PAHP) remain limited. We report on the education, employment status, and health outcomes of PAHP attending two tertiary care centers in Bangkok.

**Methods:**

PAHP ≥14 years of age attending two tertiary care centers in Bangkok were enrolled and participated in the Happy Teen 2 program (HT2), a long-term follow-up comprehensive hospital-based intervention aimed at promoting positive youth development, from June 2014 to December 2015. After completing the HT2 program, PAHP were followed up annually for an additional five years to assess their HIV care outcomes, education, and career paths. Analysis of participants’ current education, career levels, and other factors associated with obtaining a college or higher degree in year five (2020) was also conducted.

**Results:**

Of the 188 PAHP participants, 149 (79.3%) returned for follow-up. Of these, 83 (55.7%) were female, with a median age of 23.1 years; 129 (86.6%) had good literacy; 12 (8.1%) had learning disabilities; and 2 (1.3%) had physical disabilities. At the 5-year follow-up visit, 95.3% received ART, 85.9% transitioned to adult clinics, 91.1% had a CD4 count ≥200 cells/µl, and 80.0% had a viral load of <50 copies/mL. Regarding education and employment, 75.8% were employed, 30.0% were studying, and 83.0% had completed at least high school (Year 9). PAHP who were married and who consumed alcohol >3 times a week were less likely to seek a higher degree (p-value 0.036 and 0.017, respectively). Conversely, PAHP without disability and who attended HT2-individual sessions only were more likely to pursue higher education beyond Year 12 (p-value 0.005).

**Conclusions:**

Most PAHP demonstrated positive health outcomes and achieved a high level of education. Comprehensive care for PAHP that prioritizes personalized guidance and encourages higher education while limiting high alcohol consumption are essential strategies to enhance educational opportunities and career pathways as they transition into adulthood.

**Trial registration:**

This study was not registered in a clinical trial registry, as it was not designed as an interventional trial. The program was implemented as part of routine comprehensive care for adolescents with HIV at the participating clinics. This study presents observational findings related to education and career outcomes.

## Background

With broad access to anti-retroviral therapy (ART), children with perinatal HIV are living longer and transitioning into adulthood [1,2]. HIV has shifted from a life-threatening disease to a chronic health condition [3,4]. Consequently, the focus of treatment and care for individuals with HIV has moved towards enhancing their quality of life. This is particularly important for people who acquired HIV perinatally (PAHP) who have lived with HIV their entire life.

Several indicators contribute to good quality of life, with education and employment [5] being two key aspects that are closely related. Various elements influence opportunities in education and employment, including sex, familial background, cognitive abilities, health status, household income, and adolescents’ attitudes toward pursuing higher education [6–10].

PAHP are at a higher risk of experiencing impaired cognitive abilities and disrupted development [11–14]. Moreover, some PAHP often face complicated ART regimens, disease complications, and frequent hospital visits [15,16]. These challenges can contribute to inconsistent learning, interruptions in their education [17], and increased dependence on their families [18]. Stigmatization and discrimination related to HIV can negatively impact education progress, especially for those who disclose their HIV status to individuals who are not supportive of their diagnosis. Some youth living with HIV feel isolated in school environments and may experience social devaluation from their peers and family members [19,20]. Although reports on discrimination against PAHP are limited, studies indicate that children indirectly affected by HIV, such as those with infected parents or parents who died due to HIV, also face discrimination. These children may encounter discrimination and stigmatization from their classmates and even relatives, regardless of their own HIV status [21]. Such experiences can result in exclusion from educational opportunities, leading to mental health challenges, reduced learning prospects, lower retention rates, and limited career options.

Data on long-term careers and education among PAHP in Thailand are limited. We report the overall education, employment status, and health outcomes of PAHP who are receiving long-term care at two tertiary care centers in Bangkok, Thailand. We also examine the factors associated with attaining a college degree or higher in this population.

## Materials and methods

Two referral centers in Bangkok, Siriraj Hospital (SR) and Queen Sirikit National Institute of Child Health (QSNICH), collaborated with the U.S. Centers for Disease Control and Prevention (CDC) Thailand office to enroll participants in a comprehensive hospital-based positive youth intervention known as “Happy Teen 2 program” (HT2). This prospective observational cohort study was reviewed and approved by the U.S. CDC Institutional Review Board (Protocol ID 6554), the Ethical Review Committees at Thailand MOPH (ID 26/2556), QSNICH (No. 61–070), and SR (COA No. Si 034/2014). The study was conducted in accordance with the Declaration of Helsinki. As previously reported [6], all youth living with HIV aged 14 years or older who were aware of their HIV status and receiving care at SI and QSNICH between 1 March 2014 and 31 May 2014 were invited to participate in the 18-month HT2 program. PAHP’s caretakers and individuals 18 and older completed the written informed consent process. Those under 18 provided written informed assent. PAHPs and caretakers who chose not to participate in the program received regular services during their clinic visits. Participants who previously attended the initial phase of the Happy Teen 1 program (HT1) for early adolescents were also eligible to participate in the HT2 program [22]. All participants in this study received a disclosure of their HIV diagnosis.

**Fig 1:**
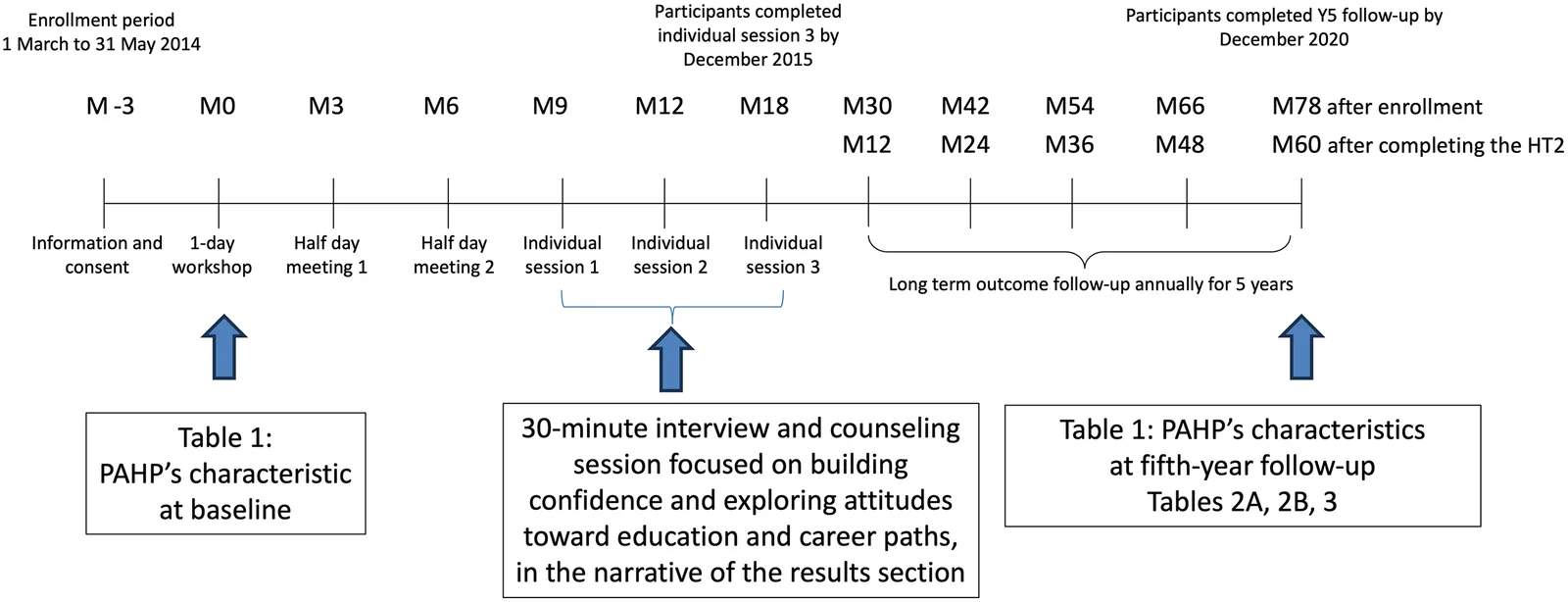
Timeline of the Happy Teen 2 interventions and annual follow-up sessions, Thailand, 2014–2020. Abbreviations: HT2, Happy Teen 2; M, Month; PAHP, People who Acquired HIV Perinatally; Y, Year.

After enrolling in the program (Fig 1), participants were interviewed using semi-structured questionnaires to assess their baseline demographic information, treatment history, knowledge, confidence, and attitudes regarding careers and education at Month 0 (M0). They were asked to complete Rosenberg’s Self-esteem scale [23], ranging from 0–30. Scores between 15 and 25 were considered normal, while scores below 15 suggested low self-esteem. Healthcare providers assessed literacy of the participants by observing their interactions in group and individual sessions based on their age, focusing on listening, reading fluency, writing, and speaking comprehension skills. Additionally, we engaged in discussions with caretakers and utilized a youth screening checklist to evaluate any challenges the youth faced at school. Good literacy was defined as the ability to read, write, speak, and listen effectively and describe key HIV knowledge and the importance of keeping appointments with healthcare providers after receiving the education sessions. Fair literacy was defined as a lack of fluency in reading, writing, speaking, or listening at a standard below their age as assessed by pediatric nurses. Poor literacy was defined as being unable to read, write, speak, or listen effectively. Physical disability was defined as a permanent limitation on a person’s physical functioning, mobility, dexterity, or stamina [24]. A learning disability was defined as a disorder in one or more basic learning processes that may manifest itself as an imperfect ability in certain areas of learning, such as reading, written expression, or mathematics [25]. To ensure consistent assessments across the two study sites, the counseling team at each site consists of one research nurse and three to four pediatric practitioners, including nurses, counselors, psychologists, and social workers. All assessors received uniform training and followed standardized protocols. Regular communication and calibration sessions were conducted among the counseling teams.

The education system in Thailand consists of a free compulsory 9-year school-based education, which includes primary school (Years 1–6) and junior high school (Years 7–9). After completing this stage, students and their families can choose to continue with the government-funded basic education in upper high school (Years 10–12) and pursue a bachelor’s degree in a 4-year college or university. Alternatively, students may enroll in a 3-year vocational school after completing Year 9 and have the option to continue for an additional two years to obtain a higher vocational degree.

The HT2 program included a one-day workshop, two half-day sessions, and three individual sessions aimed at enhancing health knowledge, coping skills, sexual risk reduction, and life goals among late adolescents. All youth participating in HT2 were invited to attend six sessions in 18 months. Participation in the program was voluntary and depended on their availability and interests, as well as those of their caregivers. Detailed topics of the six sessions can be found in the previous report [6] (Fig 1). The two half-day meetings included topics related to self-care, ART management, transition to adult care, health care benefits package, reproductive health, family planning, and partner disclosure. The one-day workshop and three individual sessions included topics related to career and education. The one-day workshop included a 2-hour group session on basic vocational orientation for PAHP and a group session for caretakers. This 2-hour session included a video presentation and group discussions to explore ways to achieve success, set personal goals, and understand the importance of career and life planning. This session aimed to foster positive attitudes and aspirations toward educational attainment and career paths. Participants were provided with resources for formal and informal education, information on career planning, details about qualifications for various professions, and a discussion regarding the requirement for HIV blood testing on job applications. The session also included assessments to evaluate personal aptitude and career competencies. Participants were invited to select topics with a counsellor during the three individual sessions. One of these topics included discussions about education and career plans, ensuring the youth knew their options and where to find additional information.

After completing the HT2 program in December 2015, the participating youth were asked to return for annual follow-up interviews conducted by healthcare providers and research nurses. The last interview was completed in December 2020 (5 years after completing the intervention). These interviews utilized semi-structured questionnaires to assess various factors, including ARV adherence, alcohol consumption behaviors, transition to adult HIV care outcomes, education, and careers from 2016–2020. Data on marital status and employment were collected starting from mid-2019 (year five of follow-up). We abstracted the latest adherence, CD4, and viral load data from the medical records at baseline. During the annual interview, we also collected CD4 and viral load data for participants who transitioned to adult care at other sites from the National AIDS program database. We analyzed data from the baseline demographic of PAHP aged 20 years or older in 2020, who completed their fifth-year annual follow-up to define their current education and career status.

### Data collection and analysis

Data managers employed double data entry to input information into a Microsoft Access database. We collected baseline and follow-up data from participants’ medical records regarding ART, ART adherence, CD4 counts, and viral load (within six months). The analysis was conducted using R software (version 4.4.2; R Core Team 2024). We analyzed demographic characteristics, careers, and education of PAHP, presented in both numbers (N) and proportions (percentages) at baseline and follow-up. We calculated the median scores on confidence and attitudes towards the careers and education of PAHP after attending the HT2 program. Factors associated with adolescents who pursued education beyond Year 12—defined as attending college/university or obtaining an equivalent or higher degree—were analyzed using logistic regression to estimate odds ratios (OR) with 95% confidence intervals (CI). Variables with a P-value less than 0.10 in bivariate analysis were subsequently included in the multivariate analysis. Unless otherwise specified, variables were obtained from baseline assessments. Variables measured at the 5-year follow-up were included to evaluate factors associated with educational attainment at the time of outcome assessment.

## Results

**Fig 2:**
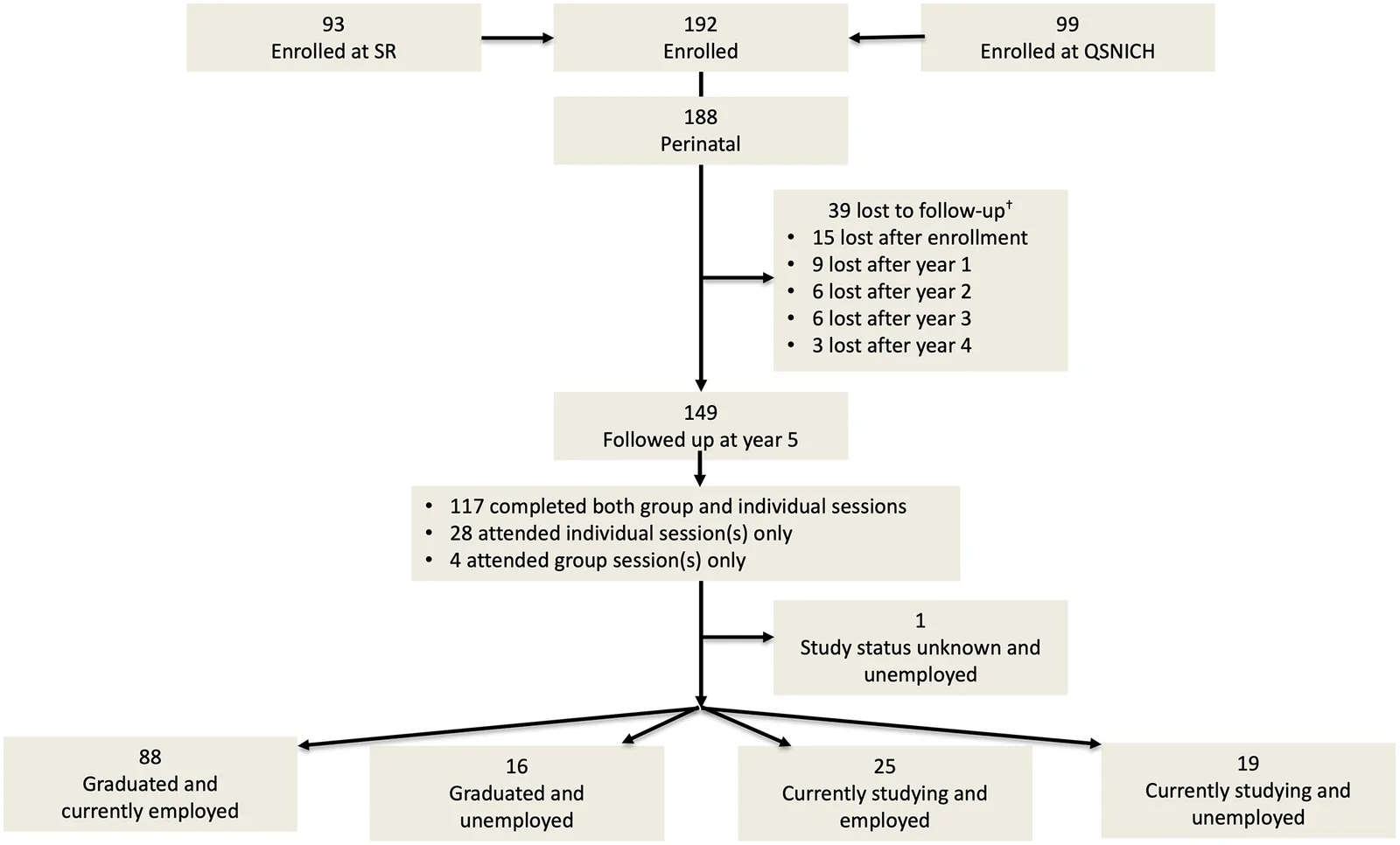
Participant flowchart for the Happy Teen 2 program. Of the 262 participants, 245 (94%) youth at the study sites met eligible criteria (age 14-21 and disclosed HIV status) and were invited to participate in the program. One-hundred ninety-two participants consented to participate in the HT2. Abbreviations: QSNICH, Queen Sirikit National Institute of Child Health; SR, Siriraj Hospital. ^†^Research nurses contacted PAHP or their caretakers on the day of missed visits and at 3, 7 days, and 1 month. Patients were considered lost to follow-up if not reached after one year. Counseling teams sometimes conducted home visits for those lost to follow-up.

In 2014, 245 of 262 (94%) youth at the study sites met eligibility criteria and were invited to participate in the program. Of these, 192 (78%) completed the consent process and were interviewed at baseline. Most, 188 (97.9%), of the 192 youth living with HIV were PAHP. Of all PAHP enrolled in HT2, 149 (79.3%) returned for the annual follow-up interview in the fifth year. Thirty-nine PAHP missed their follow-up interviews and were excluded from the data analysis (Fig 2). These 39 PAHP were similar in age and sex to those 149 included in this study. However, they had a higher percentage of parental deaths, were more likely to live with relatives or non-family members, had lower monthly incomes, and were more likely to have an unsuppressed viral load compared to the PAHP included in this study (data not shown). The median age of PAHP at enrolment to the HT2 was 16.6 years, and at the time of the 2020 follow-up was 23.1 years (Table 1).

**Table 1:** Characteristics of perinatally youth living with HIV at enrolment and fifth-year follow-up, Happy Teen 2 program, Bangkok, Thailand, 2014-2020.

Characteristics	At baseline	At fifth-year follow-up
	N (%)	N (%)
	(n=149)	(n=149)
**Age (years)**		
Median (IQR)	16.6 (15.3-18.6)	23.1 (21.9-25.1)
<15	35 (23.5)	0 (0)
15-19	95 (63.8)	0 (0)
>20	19 (12.8)	149 (100)
Sex		
Male	66 (44.3)	
Female	83 (55.7)	
***Marital Status***		
Single		109 (73.2)
Married, living together		36 (24.2)
Divorced/separated		4 (2.7)
**Hospital**		
Queen Sirikit National Institute of Child Health	71 (47.7)	
Siriraj Hospital	78 (52.3)	
**Literacy**		
Good	129 (86.6)	
Fair	17 (11.4)	
Illiterate	3 (2.0)	
**Disability**		
Physical	2 (1.3)	
Learning	12 (8.1)	
None	135 (90.6)	
**Main Caretaker(s)**		
Biological parents	72 (48.3)	
Relative & others	71 (47.7)	
Self-care	6 (4.0)	
**Currently residing with**		
Parents/caretaker/relatives	136 (91.3)	121 (81.2)
Living alone	6 (4.0)	5 (3.4)
Friends	6 (4.0)	2 (1.3)
Community residence/others	1 (0.7)	21 (14.1)
**Parental Status**		
Both alive and live together	20 (13.6)	
Both are alive but separated	14 (9.5)	
Both dead	32 (21.8)	
One of the parents dead	81 (55.1)	
Missing	2	
**Monthly household income**		
Less than 10,000 THB (<$294)	48 (32.2)	67 (45.3)
10,000-20,000 THB ($294-$588)	66 (44.3)	72 (48.6)
More than 20,000 THB (>$588)	35 (23.5)	9 (6.1)
Missing		1
**Average self-esteem score (Rosenberg)** ***Median (IQR)***	15 (14.0-16.0)	
<15	45 (30.2)	
15-25	104 (69.8)	
>25	0 (0)	
**Alcohol consumption**		
None		78 (52.3)
less than three times per week		64 (43.0)
more than or equal to three times per week		7 (4.7)
**Highest degree attained**		
Primary school (Y 6)		7 (4.7)
High school		35 (23.6)
• Junior (Y 9)		• 18 (12.2)
• Upper (Y 12)		• 16 (10.8)
• Unknown		• 1 (0.7%)
Vocational school		30 (20.3%)
• Vocational certificate^$^		• 8 (5.4)
• Higher vocation certificate		• 21 (14.2)
• Not specified		• 1 (0.7)
Undergraduate school		56 (37.8)
Graduate school		13 (8.8)
Non-formal education system$$		7 (4.7)
Missing		1
**Studying status**		
Not currently studying		104 (70%)
PAHP currently studying		44 (30)
Missing		1
**Employment status**		
Currently employed		113 (76%)
Currently unemployed		35 (24%)
Missing		1
**Time from disclosure to assessment: *Median (IQR) (months)***	55.1 (34.7-84.7)	
**Duration of receiving ART Median (IQR) (years)** ^**†**^	13.0 (10.0-15.0)	
**Age at ART initiation (years)**		
Median (IQR)	4.9 (1.2-8.0)	
<1 yr	29 (19.5)	
1-<2 yrs	17 (11.4)	
2-4 yrs	31 (20.8)	
5-9 yrs	52 (34.9)	
10-14 yrs	20 (13.4)	
**Current ART regimen**		
NNRTI-based HAART	67 (45.0)	34 (22.8)
PI-based HAART	66 (44.3)	82 (55.0)
NNRTI+PI-based regimen/ others	16 (10.7)	24 (16.1)
No ART		7 (5.7)
Taking ART but did not report the regimen		2 (1.3)
**Taking ARV during the past three days**		
Yes		136 (91.3)
No		5 (3.5)
Don’t know		3 (201)
**Transition to adult HIV care service**		
Yes		128 (85.9)
No		21 (14.1)
**CD4 count**		
< 200 cells/µl	16 (10.7)	11 (8.9)
200 - 499 cells/µl	39 (26.2)	23 (18.5)
≥ 500 cells/µl	94 (63.1)	90 (72.6)
Missing		25
**Viral load**		
< 50 copies/ml	117 (79.1)	104 (80.0)
≥50 copies/ml	31 (20.9)	26 (20.0)
Missing	1	19
**Participation in the Happy Teen 2 program** ^**‡**^		
Participated in the individual sessions	145 (97.3)	
• Participated in three individual sessions	• 122 (81.9)	
• Participated in two individual sessions	• 20 (13.4)	
• Participated in one session	• 3 (2.0)	
Participated in at least one group session	121 (81.2)	
• Participated in half-day meeting I	• 99 (66.4)	
• Participated in half-day meeting II	• 98 (65.8)	
• Participated in a one-day workshop	• 71 (47.7)	
Participated in both group and individual sessions	117 (78.5)	

†Defined as the time from ART initiation to the most recent date of receiving antiretroviral therapy during the interview visit in 2020. ^‡^Defined as attending at least one session of either individual or group sessions. Abbreviations: ARV, Antiretroviral drugs; ART, Antiretroviral therapy; CD4, Cluster of differentiation 4; HAART, Highly active antiretroviral therapy; HIV, Human immunodeficiency virus; IQR, Interquartile range; NNRTI, Non-nucleoside reverse transcriptase inhibitor; PAHP, People who acquired HIV perinatally; PI, Protease inhibitor; THB, Thai Baht; Y, Year.

At enrolment to the HT2 in 2014, 83 (55.7%) of the 149 PAHP were female, 129 (86.6%) had good literacy, and most PAHP (136, 91.3%) lived with their biological parents, caretakers, or relatives. Two PAHP had a physical disability (one with cerebral palsy and one with visual impairment) (1.3%), and 12 (8.1%) had a learning disability. About three-fourths of PAHP had lost at least one of their parents, including 32 (21.8%) PAHP had lost both parents. Only one-fourth (35, 23.5%) of PAHP had monthly household income more than the national average of $588 or 20,000 baht ($1 = 34 baht), and about one-third had a meager monthly household income of <$294 (<10,000 baht, minimal wage per month). About one-third (45, 30.2%) of PAHP had Rosenberg’s score less than 15, indicating low self-esteem. From the medical records at enrolment, all participants received ART with a median age at ART initiation of 4.9 years and a median duration of ART of 13 years. Two-thirds of PAHP had a CD4 count >500 cells/µl, with 16 (10.7%) having a CD4 count of less than 200 cells/µl. The majority 128 (86.5%) PAHP had a viral load of <1000 copies/mL (data not shown), including 117, (79.1%) had a viral load of <50 copies/mL (Table 1). Nearly all participants attended at least one HT2 individual session (145, 97.3%), and 121 (81.2%) attended at least one group session.

At the end of HT2 sessions (18 months after enrolment), with a median age of 18.1 years, youth reported improved knowledge of possible education options, their interests in the studying field, and their capability of acquiring knowledge and skills required for further careers [6]. Of the 149 PAHP, 104 (69.8%) reported strongly agreeing to have the highest education possible, 142 (95.3%) PAHP reported that they knew their education options, 147 (98.7%) knew which education or career path they were interested in, and 143 (96.0%) could choose the occupation they wanted and acquire the knowledge and skills needed for the career (data not shown in the table).

Among the 40 participants who married between 2016 and 2020, a 2020 follow-up visit revealed that, five years after completing the HT2 sessions, 36 (24.2%) PAHP were married or living with partners. This included 11 participants who married in 2020; however, 4 (2.7%) had been divorced. Of those who married in 2020, 7 (64%) participants had left school before marriage, while 4 (36%) were still enrolled in school at the time of their marriage. Additionally, 121 (81.2%) PAHP were living with their parents, caretakers, or relatives, and 67 (45.3%) PAHP had a low monthly household income of <$294. Additionally, nearly half (47.3%) of PAHP reported using alcohol, with 7 (4.7%) indicating they consumed alcohol more than three days a week. At 5-year follow up, 142 (95.3%) were receiving ART, 136 (91.3%) had taken ART on time during the past three days, and 128 (85.9%) PAHP had transitioned to adult HIV care services. Of these, 62 (48%) did so because they were over 18 years old, while another 62 (48%) transitioned due to employment changes, which required them to shift their health coverage scheme from universal health coverage to a social security scheme. For those who had not yet transitioned to adult HIV care services, 16 (76%) were in the process of preparing for the transition, 3 (14%) PAHP felt a strong connection with the clinic staff and chose not to transition, while others cited reasons such as participating in a research project (data not shown in the table). From the available medical records, 113 (91.1%) of PAHP had a CD4 count ≥ 200 cells/µl, and 104 (80.0%) had a viral load of <50 copies/mL.

Regarding education, 122 (83.0%) of 149 PAHP completed their education beyond the government compulsory level (Year 9), and 90 (61.2%) pursued education beyond Year 12 (Table 2). The highest degrees attained among the 149 PAHP are listed in Table 3. Notably, 104 (70.0%) of the PAHP have completed their formal studies. The most common degrees earned by these individuals include high school diplomas (34, 32.7%), undergraduate degrees or bachelor’s degree (30, 28.8%), vocational school certificates (28, 26.9%), followed by primary school (Year 6) (7, 6.7%), and graduate degrees, e.g., master’s degree and doctoral degree (5, 4.8%). Of the 44 PAHP who were still studying, 19 (43.2%) were enrolled in full-time programs, while 25 (56.8%) continued their education while working. Among the working students, one was studying in junior high school, one in vocational school, thirteen in college/university or equivalent, three in a post-graduate degree, and seven were engaged in non-formal education (data not shown in the Table). Those PAHP who continued their studies were more likely to obtain higher education (Table 2). One participant, who came from an orphanage, was attending non-formal education while working to support herself by year 5. In this study, 16 PAHP were neither studying nor working.

**Table 2:** The highest level of education achieved by PAHP in 2020, categorized by study and employment status under Thai compulsory education (Y9) and government-supported free education (Y12), Happy Teen 2 program, Bangkok, Thailand, N =148.

Characteristics	Highest degree attained among PAHP, n (%)						
		Categorized by studying status in 2020			Categorized by employment status in 2020		
	All PAHP N=148 (%)	PAHP currently not studying (N=104, 70%)	PAHP currently studying^¶^ (N=44, 30%)	p-value^†^	PAHP currently employed (N=113, 76%)	PAHP currently unemployed (N=35, 24%)	p-value^†^
Thai compulsory education				0.002			0.13
Less than or equal to Thai compulsory education^‡^	25 (17.0)	24 (23.3)	1 (2.3)		22 (19.6)	3 (8.6)	
Higher than Thai compulsory education^‡^	122 (83.0)	79 (76.7)	43 (97.7)		90 (80.4)	32 (91.4)	
Unknown	1	1	0		1	0	
Government-supported free education				<0.001			0.027
Less than or equal to the government-supported free education^§^	57 (38.8)	49 (47.6)	8 (18.2)		49 (43.8)	8 (22.9)	
Higher than the government-supported free education^§^	90 (61.2)	54 (52.4)	36 (81.8)		63 (56.3)	27 (77.1)	
Unknown	1	1	0		1	0	

†Pearson’s Chi-squared test ^‡^Compulsory education in Thailand: Junior high school or Year 9. ^§^Free education supported by the Thai government: Upper high school or Year 12. ^¶^PAHP were currently studying and may pursue a higher degree following the interview visit in 2020. Abbreviations: PAHP, People who acquired HIV perinatally.

**Table 3:** Highest degree attained among PAHP categorized by studying status and employment status in 2020, Happy Teen 2 program, Bangkok, Thailand.

Highest degree attained	Overall	Studying status		Employment status	
	N = 148 (%)	Completed studying N = 104 (70%)	Currently studying^†^ N = 44 (30%)	Currently employed N = 113 (76%)	Currently unemployed^‡^ N = 35 (24%)
Primary school (Y 6)	7 (4.7)	7 (6.7)	–	7 (6.2)	–
High school	35 (23.6)	34 (32.7)	1 (2.3)	29 (25.7)	6 (17.1)
• Junior (Y 9)	18 (12.2)	17 (16.3)	1 (2.3)	15 (13.3)	3 (8.6)
• Upper (Y 12)	16 (10.8)	16 (15.4)	–	13 (11.5)	3 (8.6)
• Unknown	1 (0.7%)	1 (1.0%)	–	1 (0.9%)	–
Vocational school	30 (20.3%)	28 (26.9%)	2 (4.5%)	22 (19.5)	8 (22.9)
• Vocational certificate^§^	8 (5.4)	8 (7.7)	–	6 (5.3)	2 (5.7)
• Higher vocation certificate	21 (14.2)	19 (18.3)	2 (4.5)	15 (13.3)	6 (17.1)
• Not specified	1 (0.7)	1 (1.0)	–	1 (0.9)	–
Undergraduate school^¶^	56 (37.8)	30 (28.8)	26 (59.1)	41 (36.3)	15 (42.9)
Graduate school^#^	13 (8.8)	5 (4.8)	8 (18.2)	7 (6.2)	6 (17.1)
Non-formal education system^††^	7 (4.7)	–	7 (15.9)	7 (6.2)	–

† Of the 44 PAHP currently studying, 25 PAHP were presently employed, and 19 were full-time study. ‡ Of the 35 PAHP who were unemployed, 16 PAHP were not studying. § Technical vocational school (compatible with Y12)¶ The first level of higher education typically pursued after high school, e.g., Bachelor’s degree.^#^The next level of education pursued after completing an undergraduate degree includes Master’s and Doctoral degrees.†† Compatible Y 10–12.

Abbreviations: Y, Year.

Three-quarters (113, 75.8%) of the PAHP were employed (Table 4), while 36 (24.2%) were unemployed and not studying. The top three professions among the employed PAHP include private company employees (41, 36.3%), laborers (24, 21.2%), and retailers (17, 15%). Only one PAHP (0.9%) was a government employee. Seven (6.2%) PAHP worked in professional careers such as engineering, law, and teaching. Seven (6.2%) of the employed PAHP completed only Year 6 (Table 3). PAHP who completed their education at a level below 12th grade appeared to have a higher employment rate (>80%) than those who completed 12th grade or higher (73%) (Table 2). About 72% of working PAHP earned a monthly income more than $294. Among the 113 employed PAHP, 82 (73.2%) reported that their income was sufficient to meet their needs.

**Table 4:** Current occupation status at the fifth-year follow-up visits in 2020.

Characteristics	N (%) (n = 149)	Male (%) (n = 66)	Female (%) (n = 83)	P-value
**Employment**				0.7
• Unemployed	36 (24.2)	17 (25.8)	19 (22.9)	
• Currently employed	113 (75.8)	49 (74.2)	64 (77.1)	
**Career of employed participants**				0.4
• Private company employee	41 (36.3)	17 (34.7)	24 (37.5)	
• Laborer	24 (21.2)	9 (18.4)	15 (23.4)	
• Retailer	17 (15.0)	9 (18.4)	8 (12.5)	
• Online business/ trading	8 (7.1)	1 (2.0)	7 (10.9)	
• Professional career (i.e., engineer, lawyer, teacher, etc.)	7 (6.2)	5 (10.2)	2 (3.1)	
• Self-employed/freelance	6 (5.3)	4 (8.2)	2 (3.1)	
• Service provider (i.e., food delivery, beautician, restaurant staff, hotel staff)	6 (5.3)	3 (6.1)	3 (4.7)	
• Agriculturist	3 (2.7)	1 (2.0)	2 (3.1)	
• Government/state enterprise employee	1 (0.9)	0	1 (1.6)	
**Median monthly income of employed PAHP (IQR) Thai baht**	12,000 (9,000, 15,000)	10,000 (9,000, 15,250)	12,000 (9,950, 15,000)	>0.9
**Monthly income of employed PAHP (Thai baht)**				
• <10,000 (minimum wage, < $294)	31 (27.7)	15 (31.3)	16 (25.0)	0.5
• 10,000-20,000 ($294-$588)	76 (64.3)	28 (58.3)	44 (68.8)	
• >20,000 (> $588)	9 (8.0)	5 (10.4)	4 (6.3)	
• Unknown	1 (0.001)			
**The income is sufficient for working participants.**				0.4
• Sufficient	82 (73.2)	37 (77.1)	45 (70.3)	
• Insufficient	30 (26.8)	11 (22.9)	19 (29.7)	
• Missing	1 (0.9)			

Abbreviations: IQR, Interquartile range; PAHP, People who acquired HIV perinatally; THB, Thai Baht.

In a multivariate analysis examining factors associated with PAHP who achieved a college or university degree or equivalent, the findings revealed that PAHP who were married or cohabiting with their boyfriends or girlfriends and those consuming alcohol more than 2–3 times a week were less likely to pursue a higher degree (Table 5, Table 6). Specifically, the adjusted odds ratio (aOR) for being married or living together was 0.23 (95% confidence interval [CI], 0.09–0.58; p-value 0.003), while for alcohol consumption >2–3 times/week, the aOR was 0.05 (95% CI, 0.0–0.45; p-value 0.017) (Table 6). Conversely, PAHP without disabilities were significantly more likely to pursue higher education, with an aOR of 7.74 (95% CI, 1.79–54.3; p-value 0.005). Furthermore, those who attended only HT2-individual sessions had an even higher likelihood, with an aOR of 8.48 (95% CI, 2.09–63.2; p-value 0.005).

**Table 5:** Univariate analysis of factors associated with PAHP who attained college/university or equivalent and higher degree, high vs. low education attainment.

Factors	Education			Univariate		
	Overall N = 147	Low education N = 57	High education N = 90	OR	95% CI	p-value
**Hospital**						0.8
A	69 (46.9%)	26 (45.6%)	43 (47.8%)	—	—	
B	78 (53.1%)	31 (54.4%)	47 (52.2%)	0.92	0.47, 1.78	
**Literacy**						**0.005**
Poor or Fair reading and writing	19 (12.9%)	13 (22.8%)	6 (6.7%)	—	—	
Good reading and writing	128 (87.1%)	44 (77.2%)	84 (93.3%)	4.14	1.52, 12.5	
**Age**^†^ **(years)**						**0.039**
< 23	72 (49.0%)	34 (59.6%)	38 (42.2%)	—	—	
>= 23	75 (51.0%)	23 (40.4%)	52 (57.8%)	2.02	1.04, 4.01	
**Sex**						0.5
Male	64 (43.5%)	23 (40.4%)	41 (45.6%)	—	—	
Female	83 (56.5%)	34 (59.6%)	49 (54.4%)	0.81	0.41, 1.58	
**Currently residing with** ^†^						**0.009**
Living alone or with friends	7 (4.8%)	1 (1.8%)	6 (6.7%)	—	—	
Parents/Caretaker/relatives	119 (81.0%)	42 (73.7%)	77 (85.6%)	0.31	0.02, 1.87	
Community residence/others	21 (14.3%)	14 (24.6%)	7 (7.8%)	0.08	0.00, 0.62	
**Parental status**						0.2
Both alive and live together	20 (13.8%)	6 (10.7%)	14 (15.7%)	—	—	
Both alive but separated	14 (9.7%)	7 (12.5%)	7 (7.9%)	0.43	0.10, 1.75	
Both dead	32 (22.1%)	17 (30.4%)	15 (16.9%)	0.38	0.11, 1.20	
One of the parents dead	79 (54.5%)	26 (46.4%)	53 (59.6%)	0.87	0.28, 2.45	
Unknown	2	1	1			
**Monthly income** ^†^						0.10
< 10,000	65 (44.5%)	29 (51.8%)	36 (40.0%)	—	—	
10,000 - 20,000	72 (49.3%)	26 (46.4%)	46 (51.1%)	1.43	0.72, 2.84	
> 20,000	9 (6.2%)	1 (1.8%)	8 (8.9%)	6.44	1.09, 123	
Unknown	1	1	0			
**Age at ART initiation (years)**						>0.9
< 5	77 (52.4%)	30 (52.6%)	47 (52.2%)	0.68	0.24, 1.91	
5 – < 10	50 (34.0%)	19 (33.3%)	31 (34.4%)	0.90	0.35, 2.26	
10 – < 15	20 (13.6%)	8 (14.0%)	12 (13.3%)	0.83	0.26, 2.67	
**Patient average self-esteem score (Rosenberg)**						0.2
< 17	43 (29.9%)	13 (23.2%)	30 (34.1%)	—	—	
17 - 21	62 (43.1%)	29 (51.8%)	33 (37.5%)	0.49	0.21, 1.11	
> 21	39 (27.1%)	14 (25.0%)	25 (28.4%)	0.77	0.30, 1.95	
Unknown	3	1	2			
**Disability**						**<0.001**
Yes	14 (9.5%)	12 (21.1%)	2 (2.2%)	—	—	
No	133 (90.5%)	45 (78.9%)	88 (97.8%)	11.7	3.03, 77.5	
**Marital status** ^†^						**<0.001**
Single	108 (73.5%)	30 (52.6%)	78 (86.7%)	—	—	
Married, living together	35 (23.8%)	24 (42.1%)	11 (12.2%)	0.18	0.07, 0.40	
Divorced/separated	4 (2.7%)	3 (5.3%)	1 (1.1%)	0.13	0.01, 1.04	
**Received ART in the past three months** ^†^						**0.024**
No	7 (4.8%)	5 (9.1%)	2 (2.2%)	—	—	
Yes, NNRTI-based ART	34 (23.4%)	9 (16.4%)	25 (27.8%)	6.94	1.26, 55.0	
Yes, PI-based ART	81 (55.9%)	36 (65.5%)	45 (50.0%)	3.13	0.63, 22.7	
Yes, others	23 (15.9%)	5 (9.1%)	18 (20.0%)	9.00	1.47, 78.3	
Unknown	2	2	0			
**CD4**^†^ **(cell/μl)**						**0.020**
< 200	11 (9.0%)	7 (15.9%)	4 (5.1%)	—	—	
200 - 499	21 (17.2%)	11 (25.0%)	10 (12.8%)	1.59	0.36, 7.66	
>= 500	90 (73.8%)	26 (59.1%)	64 (82.1%)	4.31	1.20, 17.6	
Unknown	25	13	12			
**Viral load**^†^ **(copies/ml)**						**<0.001**
< 50	103 (80.5%)	30 (63.8%)	73 (90.1%)	—	—	
>= 50	25 (19.5%)	17 (36.2%)	8 (9.9%)	0.19	0.07, 0.48	
Unknown	19	10	9			
**Alcohol consumption** ^†^						**0.028**
None	76 (51.7%)	29 (50.9%)	47 (52.2%)	—	—	
Less than 2–3 times/week	64 (43.5%)	22 (38.6%)	42 (46.7%)	1.18	0.59, 2.37	
2-3 times/week or more	7 (4.8%)	6 (10.5%)	1 (1.1%)	0.10	0.01, 0.64	
**Wanting to have the highest education possible**						0.13
Less than strongly agree	51 (35.2%)	24 (42.9%)	27 (30.3%)	—	—	
Strongly agree	94 (64.8%)	32 (57.1%)	62 (69.7%)	1.72	0.86, 3.47	
Unknown	2	1	1			
**Attended HT2 sessions**						**<0.001**
Both group and individual sessions	115 (78.2%)	52 (91.2%)	63 (70.0%)	—	—	
Individual session only	28 (19.0%)	2 (3.5%)	26 (28.9%)	10.7	3.01, 68.6	
Group session only	4 (2.7%)	3 (5.3%)	1 (1.1%)	0.28	0.01, 2.22	

†Variables in the analysis were obtained from baseline data. However, for variables assessed at both baseline and the 5-year follow-up, the values from the 5-year follow-up were used in the analysis, including age, marital status, current residing with, ART regimen, alcohol consumption, CD4, and VL.

Data were presented as n (%) and odds ratio (95% CI), as appropriate. Of the 147 participants, two were excluded, leaving 145 for the multivariate analysis. Abbreviations: ART, Antiretroviral therapy; CD4, Cluster of differentiation 4; CI, Confidence interval; HT2, Happy Teen 2 program; IQR, Interquartile range; NNRTI, Non-nucleoside reverse transcriptase inhibitor; OR, Odds ratio; PAHP, People who acquired HIV perinatally; PI, Protease inhibitor.

**Table 6:** Multivariate analysis of factors associated with PAHP who attained college/university or equivalent and higher degree.

Factors	Multivariate		
	OR	95% CI	p-value
**Disability**			**0.005**
Yes	—	—	
No	7.74	1.79, 54.3	
**Marital status** ^†^			**0.003**
Single	—	—	
Married, living together	0.23	0.09, 0.58	
Divorced/separated	0.14	0.01, 1.33	
**Alcohol consumption** ^†^			**0.017**
None	—	—	
Less than 2–3 times/week	1.03	0.44, 2.45	
2-3 times/week or more	0.05	0.00, 0.45	
**Attended HT2 sessions**			**0.005**
Both group and individual sessions	—	—	
Individual session only	8.48	2.09, 63.2	
Group session only	0.61	0.02, 9.77	

† Variables in the analysis were obtained from baseline data. However, for variables assessed at both baseline and the 5-year follow-up, the values from the 5-year follow-up were used in the analysis, including marital status and alcohol consumption.

Data were presented as odds ratio (95% CI). Of the 147 participants, 145 were included for the multivariate analysis Abbreviations: CI, Confidence interval; HT2, Happy Teen 2 program; OR, Odds ratio.

## Discussion

This paper emphasizes the relationship between HT2 interventions and treatment outcomes, alongside retention in educational and career opportunities. It advocates for integrated HIV care interventions and prepares PAHP for their transition to adulthood. Despite the challenges of chronic illness, PAHP attending the HT2 program demonstrated high treatment success rates, educational retention, and employment. The HT2 program significantly enhanced their confidence and attitudes toward education and career planning. Our previous study found that most PAHP involved in the HT2 program reported an increase in their knowledge of education and career planning [6]. In our cohort, the majority (95%) of PAHP over 20 years old completed Year 9, and 83% completed Year 12 or attended a technical vocational school. These completion rates are higher than the Thai national average rate of 86% for Year 9 and 47% for Year 12, as well as the average education rates in urban areas of Thailand (90% for Year 9 and 56% for Year 12) [26].

On average, PAHP in this cohort began ART before the age of 5, and most reported having good literacy skills and adherence to their ART resulting in over 80% viral load suppression. Our findings align closely with reports from Western countries [27] and support the recommendations of the WHO and neurodevelopmental studies, which suggest that early HIV treatment leads to better health outcomes [28]. We found that individuals without disabilities participating in HT2-individual sessions were more likely to pursue higher education. While group sessions provide an efficient way to deliver standardized educational content and foster peer learning, individual sessions offer personalized guidance that caters to each participant’s educational goals, abilities, and challenges. The observed link between attendance at individual sessions only and educational achievement may indicate the added benefit of individualized support. Additionally, PAHP who attended only individual sessions appeared to represent an older and more socioeconomically advantaged subgroup. They were significantly older, had higher literacy levels, and reported higher household income than those who attended group sessions (S2 Table). They had also lived longer with knowledge of their HIV status before assessment. In contrast, clinical characteristics, including CD4 count, viral suppression, ART duration, age at ART initiation, and ART regimen, were comparable between the two groups, suggesting that differences in participation patterns may have been driven by demographic and social factors. Nevertheless, group sessions could still offer substantial benefits if structured into smaller sizes to facilitate and monitor active participation among learners. In this study, the group sessions on career and education topics were delivered as large-scale workshops (approximately 30 PAHP per group plus caretakers) conducted outside the hospital setting, primarily in a lecture format and group activities. Due to this format, youth may absorb the content differently than in the tailored, interactive messages of individual sessions, which actively assess understanding and co-develop personalized plans. Transitioning to smaller, more controlled group sizes could address these limitations, fostering higher engagement and interaction. In resource-limited settings, offering individual sessions and integrating them into routine services—alongside optimizing group session formats— may help retain participants in educational programs. Our team highlighted the importance of higher education and employment during annual clinical visits to reinforce key messages and support informed decision-making.

The Thai government provides free education for 12 years, with a compulsory 9-year period, for all Thai citizens [29]. However, students with chronic illnesses often face challenges, resulting in poorer school attendance [30], and lower levels of educational attainment [31]. These issues can impact their future career options. In our study cohort, we observed that the education retention rate among PAHP was better than the national average. Over 80% of PAHP in our cohort completed year 12, 48% continued to college or university, and 4.7% pursued post-graduate degrees (Master’s/PhD). Less than 5% of PAHP reported their highest education level as primary school, and only 12% had completed up to Year 9. This increased educational attainment may be attributed to the fact that most PAHP in this cohort live in Bangkok, which offers better educational opportunities than other regions of Thailand. Additionally, we found that some PAHP pursued studies in either the formal or informal education systems. The HT2 program, particularly the individual session, plays a role in improving the attitudes and confidence of PAHP to continue their education and plan their career paths.

Our study found that most PAHP successfully transitioned to adult HIV care services while maintaining good adherence to ART, resulting in viral load suppression. Notably, about a quarter of these individuals were either married or living with a partner. Additionally, the majority of these PAHP did not engage in heavy alcohol consumption and had stable employment. Approximately three-quarters were employed in the private sector, primarily in labor and retail positions. Most employed had completed Year 12 education, which is higher than the compulsory education requirements. More than 70% earned a monthly income that exceeded Thailand’s minimum monthly wage, which ranges from 328–354 THB ($9.6–10.4) per day or 10,000 THB ($294) per month (32). Their income was close to the average monthly wage for the general Thai population, which is around 15,412 THB ($453.3) per month (33). This finding contrasts a study conducted in India, where PAHP reported lower levels of education, occupation status, and income [32].

Among the participants, seven PAHP indicated that their highest level of education was primary school, yet all were employed. Out of these seven, two (28.5%) had fair literacy, which could hinder their ability to pursue higher education. Furthermore, six (85.7%) of these seven individuals had a monthly household income below the minimum wage, which may have compelled them to leave school early to support their families. None of these seven patients returned to continue their education. PAHP who have less than a 12th-grade education or who have gone through a non-formal education system tend to have higher employment rates compared to those with a 12th-grade education or higher. This trend may be attributed to the fact that some PAHP participants had to leave their education to work due to socioeconomic challenges, while others chose to pursue further studies after completing 12th grade instead of entering the workforce immediately.

The correlation between limited education, restricted career opportunities, and lower household income was evident in the follow-up questionnaire, which showed a lower average monthly income among these participants. Factors such as lower education levels, fewer opportunities, poor literacy, restricted career choices, and decreased income are interconnected. Additionally, the challenges of HIV-related stigma, health issues, and the burden of treatment may exacerbate these circumstances. To break this cycle and enhance overall outcomes, it is essential to incorporate interventions that improve educational opportunities within comprehensive HIV care and counseling. Healthcare providers should stress the significance of early ART and encourage PAHP to pursue further education. This approach aligns with the Thai government’s initiative to expand free education, thereby enhancing career paths and employment opportunities.

Although the Department of Labor Protection and Welfare in Thailand advocates for the elimination of HIV blood testing before employment [33], some private companies and government organizations still require such testing as a condition of hiring. This study shows that most PAHP prefer to work in private companies or pursue their own businesses. Only a small number (eight) of PAHP were employed in government positions or professional careers. However, we documented instances of employment barriers related to HIV status, as some participants reported being rejected from jobs or leaving positions—particularly in the hospitality sector—when pre-employment or post-probation medical examinations, including blood testing, were mandated. Both adolescents and their caregivers expressed concerns about the involuntary disclosure of HIV status, which may influence career choices and restrict employment opportunities. Equal employment opportunities for PAHP play a significant role in fostering inclusivity and empowering PAHP to reach their full potential.

Our study had certain limitations. The population we studied consisted of PAHP receiving care at pediatric tertiary care centers in Bangkok, who returned for regular follow-up visits. These PAHP were part of the HT2 program, which provided essential education on HIV along with guidance on life skills, career planning, and educational goals through multidisciplinary teams led by pediatric HIV specialists. As a result, our findings may not apply to PAHP receiving care in clinics that do not offer comprehensive counseling and support or to those with varying parental statuses and socioeconomic backgrounds. Additionally, we lacked data on the educational attainment and employment of youth without HIV, and PAHP who did not attend HT2, which could have served as a control group for our study. However, we did compare the education levels, average monthly wages, and the ability to secure a career that generates income comparable to the Thai average. Data on marital status and employment were collected beginning at the fifth-year follow-up, limiting our ability to determine the temporal sequence between school completion, workforce entry, and marriage. Alcohol consumption patterns among adolescents have varied over the years. Before 2020, twenty-one individuals reported using alcohol 2–3 times per week, but most reduced their intake by 2020, except for four who maintained their usage. Three drank less frequently but increased their use in 2020. This emphasizes the need for ongoing assessment of adolescent substance use. Our study did not measure the quantity consumed, binge drinking, or alcohol use disorder symptoms, which limits our evaluation of severity. Notably, no adolescents reported daily alcohol consumption. Furthermore, the COVID-19 pandemic disrupted education and career opportunities in the general population, likely affecting PAHP as well. Consequently, this study may not be generalizable to the post-COVID era.

In conclusion, most PAHP in this study demonstrated positive health outcomes, strong educational retention, and stable employment, with nearly two-thirds either employed or running their own businesses, earning wages comparable to the average in Thailand. Early initiation of ART in infants with HIV and continuous adherence to treatment significantly improve health and quality of life. Evaluating attitudes towards education can highlight growth areas, while promoting a positive outlook on living with HIV and moderating alcohol consumption can improve overall well-being. Discussing career paths and access to higher education during clinic visits can empower PAHPs and support their future success. Ongoing follow-up can help monitor career progression and assess mental health and social relationships, identifying areas that require additional support.

## Supporting information

S1 Dataset Minimal dataset of PAHP cases in the Happy Teen 2 program. (CSV)

S2 Table Characteristics of the 28 PAHP who attended only individual sessions compared to the 121 PAHP who attended group sessions. (DOCX)
