# Supplementary material for: Career and education outcomes among people who acquired HIV perinatally and received care at two tertiary care hospitals in Bangkok, 2014–2020
Source: PLoS One. 2026 Aug 25;21(8):e0348339. doi: 10.1371/journal.pone.0348339 (PMC13505903; doi:10.1371/journal.pone.0348339)
Supplement: Supplementary file 1 [file pone.0348339.s002.docx]

**S2 Table. Characteristics of the 28 PAHP who attended only individual sessions compared to the 121 PAHP who attended group sessions**

| Baseline characteristics  at baseline | N = 28*^1^* | N = 121 | P-value |
| --- | --- | --- | --- |
| Age (years) at enrollment |  |  | <0.001 |
| Median (Q1, Q3) | 18.5 (17.6, 20.2) | 16.3 (14.9, 17.9) |  |
| Age group at enrollment |  |  | 0.024 |
| < 15 | 3 (10.7%) | 31 (26.5%) |  |
| 15 - 19 | 18 (64.3%) | 76 (65.0%) |  |
| >= 20 | 7 (25.0%) | 10 (8.5%) |  |
| Gender |  |  | 0.14 |
| Male | 16 (57.1%) | 49 (41.9%) |  |
| Female | 12 (42.9%) | 68 (58.1%) |  |
| Literacy |  |  | 0.044 |
| Good reading and writing | 28 (100.0%) | 97 (82.9%) |  |
| Fair reading and writing | 0 (0.0%) | 17 (14.5%) |  |
| Poor reading and writing | 0 (0.0%) | 3 (2.6%) |  |
| Main caretaker(s) |  |  | 0.3 |
| Parent(s) | 17 (60.7%) | 54 (46.2%) |  |
| Relatives & others | 11 (39.3%) | 58 (49.6%) |  |
| Self-care | 0 (0.0%) | 5 (4.3%) |  |
| Parental status |  |  | 0.7 |
| Both alive and live together | 4 (14.3%) | 16 (13.9%) |  |
| Both alive but separated | 2 (7.1%) | 12 (10.4%) |  |
| Both dead | 4 (14.3%) | 27 (23.5%) |  |
| One of the parents dead | 18 (64.3%) | 60 (52.2%) |  |
| Monthly income at enrollment |  |  | 0.004 |
| < 10,000 | 3 (10.7%) | 45 (38.5%) |  |
| 10,000 - 20,000 | 13 (46.4%) | 50 (42.7%) |  |
| > 20,000 | 12 (42.9%) | 22 (18.8%) |  |
| Time from disclosure to assessment (months) | 65.8 (53.3, 85.7) | 50.9 (32.0, 84.9) | 0.021 |
| Duration of receiving ART (years) | 13.5 (11.0, 16.0) | 13.0 (10.0, 15.0) | 0.11 |
| CD4 (cell/μl) |  |  | >0.9 |
| < 200 | 3 (10.7%) | 12 (10.3%) |  |
| 200 - 499 | 8 (28.6%) | 30 (25.6%) |  |
| >= 500 | 17 (60.7%) | 75 (64.1%) |  |
| Viral load (copies/ml) |  |  | 0.4 |
| < 50 | 20 (74.1%) | 95 (81.2%) |  |
| >= 50 | 7 (25.9%) | 22 (18.8%) |  |
| Age at ART initiation (years) |  |  | 0.5 |
| Median (Q1, Q3) | 5.2 (1.7, 8.3) | 4.9 (1.2, 7.7) |  |
| Age group at ART initiation |  |  | 0.9 |
| < 1 | 4 (14.3%) | 26 (22.2%) |  |
| 1 - < 2 | 4 (14.3%) | 13 (11.1%) |  |
| 2 - < 5 | 6 (21.4%) | 22 (18.8%) |  |
| 5 - < 10 | 11 (39.3%) | 40 (34.2%) |  |
| 10 - < 15 | 3 (10.7%) | 16 (13.7%) |  |
| Current ART regimen |  |  | 0.6 |
| NNRTI-based regimen | 15 (53.6%) | 51 (43.6%) |  |
| PI-based regimen | 12 (42.9%) | 52 (44.4%) |  |
| NNRTI+PI-based regimen | 1 (3.6%) | 13 (11.1%) |  |
| Others | 0 (0.0%) | 1 (0.9%) |  |
